# Feasibility of Self-Management of Hypertension and Diabetes Using Patient-Generated Health Data Through M-health in Central India

**DOI:** 10.7759/cureus.55060

**Published:** 2024-02-27

**Authors:** Aviraj K S, Surya Bali, Abhijit P Pakhare, Sagar Khadanga

**Affiliations:** 1 Community and Family Medicine, All India Institute of Medical Sciences, Bhopal, Bhopal, IND; 2 Internal Medicine, All India Institute of Medical Sciences, Bhopal, Bhopal, IND

**Keywords:** self-care management, patient-generated health data, hypertension, diabetes, mhealth, telehealth

## Abstract

Introduction: Digital health innovations are modern solutions for the management and treatment of diseases, particularly non-communicable diseases. This study was conducted to assess the feasibility of the management of diabetes and hypertension through patient-generated health data (PGHD).

Methods: A mobile application (One Health) was used for the entry of data on blood pressure, blood glucose, physical activity, and dietary intake for six months by the patients. Frequency of application download, monthly data entry, and change in control of blood pressure and glycemic markers during six months of application use were used to assess the feasibility and impact of the application for the management of hypertension and type 2 diabetes mellitus (T2DM).

Results: A higher proportion of patients entered data generated non-invasively (blood pressure) as compared to data generated via invasive techniques (glycemic control measures). The frequency of monthly data entry remained unchanged during One Health use. At the end of six months, control of hypertension and glycemic control was achieved in 53.3% and 33.9% of patients, respectively. Furthermore, there was a significant reduction of mean diastolic blood pressure (0.60 (±2.31); p=0.03) and glycosylated hemoglobin (0.657 (±1.808); p=0.04).

Discussion: The acceptability of One Health appears to be associated with the frequency of data entry. The entry of health data by patients was sufficient to effect positive change in health indicators. Although mobile applications enable the monitoring and management of chronic health conditions, additional research shall help in optimizing interventions to be adopted as acceptable tools of patient management.

## Introduction

The burden of non-communicable diseases (NCDs) has steadily increased from 1990-2019, globally [1]. Package of Essential Non-Communicable (PEN) Disease Intervention by the World Health Organization (WHO) advocates the integration of NCD management services in the Primary Health Center (PHC) [2]. This step has demonstrated improved management of NCDs in resource-limited community settings [3,4].

Information and Communication Technologies are used for managing illnesses, and health risks, and promoting well-being, termed Digital Health (DH) [5]. With the advent of Artificial Intelligence DH will become an unavoidable and dominant theme in healthcare [6]. Although such technologies are associated with strengths and limitations, wearable healthcare devices and supporting mobile health applications are now accepted in some healthcare models [7]. DH is being termed a paradigm shift [8] and is acceptable and beneficial to patients even without prior sensitization [9,10]. Internet-enabled mobile communication devices have facilitated patients to collect and record data on their health [11]. This has been termed patient-generated health data (PGHD), including medical history and symptoms and recorded and inferred medical data [12]. PGHD has been widely used in clinical, in-patient, and out-patient settings [13]. 58.3% of United States citizens have downloaded at least One Health-related mobile application. 45.7% have discontinued its use due to various reasons [14]. The use of mobile technology in promoting health has opportunities and challenges. The latter includes addressing barriers and drivers of health behaviors, high PGHD entry load, and loss of interest [14,15].

## Materials and methods

To assess feasibility, a prospective longitudinal study was conducted for 14 months, from May 2020 to July 2021. Ethics approval for the study was obtained from the Institutional Human Ethics Committee-Post Graduate Research (LOP/2020/MD0103). Participant recruitment was done in the ambulatory clinic of General Medicine (GM) or Community and Family Medicine (CFM) departments of AIIMS-Bhopal. Identification of prospective participants was done through purposive sampling. Recruitment was done through convenience sampling. Patients who agreed to participate in the first attempt were included. The eligibility criteria for participants are given in Table 1.

**Table 1:** Eligibility criteria for participation in the study

Inclusion	Exclusion
18 years of age and above	No access to internet-enabled mobile phone
Able to speak and read English/Hindi.	Not understanding either Hindi or English
Cases of Type II Diabetes Mellitus and Hypertension diagnosed for at least three months.	Patients recently diagnosed with Type II Diabetes and Hypertension (Less than three months).

Mobile application-based PGHD collection

The mobile-based application was the primary data collection tool. The application was termed One Health. After brainstorming blood pressure, blood glucose, physical activity, diet, and medication data were identified as domains to be assessed through the application.

The development of One Health was outsourced. Frequent discussions with the app developers ensured the capture of required data. All domains of One Health were individually tested at each stage to allow identification and rectification of any error. One Health was solely developed for the Android platform and was launched after three months of constant discussions and revisions. Following its launch in Google Playstore, One Health was pilot-tested by 30 participants to identify errors in execution and PGHD collection.

Assessment of the feasibility of One Health use

For the assessment of the application, the assessment of five key variables was considered crucial. These included pharmacological treatment for the health condition, blood pressure measurements, blood glucose measurement-fasting, post-prandial, calorie intake-daily calorie intake in major and minor meals, and physical activity-duration of daily physical activity. Data on these variables were entered by the patients in the mobile application for six months. The data were further analyzed for the results.

Four variables were used as indicators for the assessment of the feasibility of usage, namely, completeness of PGHD entry at registration, completeness of follow-up, completeness of PGHD entry at each follow-up, and status of control of clinical parameters at baseline and end-line. A detailed description of monitoring and variables is available in supplementary data.

Implementation

Recruited patients were briefed regarding self-monitoring practices. The process of registration on One Health was explained. Patients and/or caregivers were provided psycho-motor training and instructions for entering and uploading required data. Videographic resources and a telephone helpline were also made available. Data entered in One Health were synchronized with the server. This PGHD is from registered participants and was downloaded after six months.

Statistical analysis

Data entered by the patient in One Health was exported to an Excel spreadsheet (Microsoft Excel, Microsoft 365, USA). Data was cleaned for ambiguity and identification/rectification of outliers. cleaned data was imported in RStudio IDE for wrangling and subsequent analysis in RStudio (v 4.2) using packages, namely, tidyverse, readxl, lubridate, and their dependencies. Multiple data points for all the variables were linked using the which generates identifiers unique for all participants. Multiple data points for blood pressure, blood glucose, and physical activity were reduced to weekly data points. Daily data for diet and medication was used as is.

Data were imported into Statistical Package for Social Sciences (SPSS) version 26 (IBM Corp., Armonk, NY, USA) for further analysis. A month was considered as the time unit and change in parameters in unit time (monthly) was assessed. The data at baseline and end-line were compared. A full plan of analysis is discussed in Supplementary data.

## Results

The study was conducted for six months. 500 patients were screened in GM and CFM OPDs. 298 patients agreed to participate and completed the process of registration. 177 (59.4%) were males and 121 (40.60%) were females. The mean age of the participants was 51.94±12.95 years (49.80±13.24 for females and 53.40±12.58 for males) (Table 2). Of the registered participants, 228 participants completed the study for six months, whereas 68 participants were lost to follow-ups.

**Table 2:** Description of the study sample

Characteristic	Overall, N = 298	Female, N = 121	Male, N = 177
Age	51.94±12.95	49.80±13.24	53.40±12.58
Weight in kg	67.45±12.23	64.81±11.50	69.27±12.42
Height in cm	247.44±1,048.91	160.48±8.88	306.88±1,359.33
BMI	25.26±5.16	25.33±5.11	25.21±5.20
Daily activity			
Heavy	36 (12.08%)	18 (14.88%)	18 (10.17%)
Moderate	77 (25.84%)	32 (26.45%)	45 (25.42%)
Sedentary	185 (62.08%)	71 (58.68%)	114 (64.41%)

Download and registration details of the One Health app

The total number of One Health downloads and registrations was 295. One hundred and ninety-six participants registered during the first month of the launch of One Health (May 2020), whereas 81 (27.36%) registrations occurred in the second month of launch (June 2020), and the remaining 19 participants registered in subsequent months (Table 3).

**Table 3:** Time of registration

S. No.	Variable	Number of downloads (n, %)
1	May	196 (66.21%)
2	June	81 (27.36%)
3	July onwards	19 (6.41%)

Completeness of PGHD on registration

Two hundred and eighty-seven participants completed data entry on registration. Blood pressure data were entered by 149 participants (50.5%), whereas 223 (77.7%) participants entered data for blood sugar (Table 4).

**Table 4:** Completeness of PGHD entry at registration (n=287)

S. No.	Variable	Number of completed entries (n, %)
1	Blood Pressure	149 (50.5)
2	Blood Glucose	223 (77.7)
3	HbA1C	36 (12.54)
4	Diet	48 (16.4)
5	Physical Activity	40 (13.7)

Completeness of follow-ups

Out of 298 participants initially registered, 228 (77.3%) participants continued entering the data into One Health. Other participants were considered lost to follow-up. From the 228 participants who continued PGHD entry after registration, seven participants (3.1%) did not enter data after the first month whereas, 221 (96.9%) participants continued and completed the data entry for six months.

Completeness of PGHD at follow-ups

Of the 228 participants who entered the data at multiple time points, 138 (60.5%) had complete data with all six-monthly follow-ups. Ninety participants (39.5%) had incomplete data in at least one of the follow-ups.

Monthly PGHD entry

The mean frequency of monthly data entries was highest in the month of registration and the fifth month after registration (213 and 214, respectively). It was lowest in the month following registration. The mean frequency of data entries during the study was 211 (rounded off to the nearest digit).

Status of control

Table 5 shows the control status of blood pressure, blood glucose, and HbA1c at baseline and end-line. Eighty patients (53.3%) had controlled blood pressure (systolic and diastolic) at the end-line as opposed to 70 patients (46.7%) at baseline. Similarly, 71 patients (33.9%) had controlled blood glucose levels (fasting and post-prandial, both) at the end-line as opposed to 64 patients (30.2%) at baseline. Three patients (of 67 patients who entered data at end-line; 4.47%) had controlled HbA1c as compared to two patients (from 71, 2.8%) at baseline.

**Table 5:** Control status of BP, BG and HbA1c at baseline and end-line

Status	Baseline, n (%)	End-line, n (%)
BP uncontrolled	80 (53.3)	70 (46.7)
BP controlled	70 (46.7)	80 (53.3)
BG uncontrolled	148 (69.8)	141 (66.5)
BG controlled	64 (30.2)	71 (33.9)
HbA1C uncontrolled	70 (97.2)	64 (94.2)
HbA1C controlled	2 (2.8)	3 (4.47)

Frequency of monthly data entry

The frequency of monthly data entry was assessed. The number of times a participant has entered PGHD was assessed and categorised as no entry, random entry, once a month, twice a month, thrice a month, four times a month, and more than four times a month. Most of the participants (85; 37.3%) entered the data twice a month for a total of 12 times in six months. Seventy-seven participants entered the data 24 times during the study. Twenty-three participants (10.08%) entered the data daily for six months.

Effect of One Health

The mean systolic and diastolic blood pressure for the participants who completed data entry for six months was 131.41 (±7.14) mm Hg and 77.50 (±6.54) mm Hg, respectively, at the baseline. Mean systolic and diastolic BP was 131.15 (±8.22) mm Hg and 76.90 (±6.01) mm Hg, respectively at the end-line. The mean drops in systolic and diastolic BPs were found to be -0.25 (±7.95) mm Hg and -0.60 (±2.31) mm Hg, respectively. The difference between baseline and end-line values of DBP was statistically significant (p=0.033). The difference in the baseline and end-line values of SBP was not statistically significant.

Similarly, the mean HbA1C of the participants who completed PGHD entry was 9.78 (±1.45) at the baseline compared to 9.11 (±1.44) at the end-line. The mean drop in the HbA1c was found to be 0.657 (±1.808). This reduction in mean HbA1c was found to be statistically significant with p=0.042.

## Discussion

In this paper, the development of a mobile application for monitoring general health and lifestyle practices of patients with hypertension and diabetes, who are on hospital-based pharmacological treatment is reported. It was attempted to determine the number of patients willing to download and use such applications and the frequency of use and usage as intended.

Patient profile

The mean age was 51.94 years (12.95) and 59.4% were males. Quinn et al. [16] studied the efficacy of diabetes management mobile applications in older individuals (mean age 70.3 years). They conducted the study in a representative sample of seven individuals of which 57.1% were females. It is too small a number to be quoted.

Zhai and Yu conducted a hospital study of 60 patients with a mean age of 54.12 years with 50.2% males [17]. The mean BMI was 26.41 (4.41%), and the baseline HbA1c was 8.16 (2.57%). The mean BMI in this study at baseline was 25.26 (5.16%). Bengtson et al. [18] conducted their study on nine women and 12 men suffering from hypertension and reported a median age of 69 years and 62.5 years, respectively.

Willingness to use One Health

About 60% of the approached patients agreed to participate in the study. Paradis et al. reported in a cross-sectional study that 47.7% of citizens of France use at least one health-based mobile application, and 50% use applications related to medication, treatment, and follow-up [19]. Liu et al. have reported that 81/108 (75%) and 50/52 (96.15%) of low-income patients attending primary care facilities showed interest in using mobile-based health applications when aided by a community health worker (CHW) [20]. This study also conducted in a primary care setting, did not employ CHWs. People with chronic diseases are willing to use mobile application-based health monitoring strategies, and such usage can be enhanced with the involvement of patient-trusted stakeholders. Younger age increases the utilization of mobile-based health applications usage., The prevalence of an older age group (61-70 years) perhaps implies more acceptance or willingness to use such applications in India. Chronic disease status significantly increases the usage of mobile-based health applications [21]; also established in this study.

Continued use of One Health

User experience and intent have been identified as key modulators of consistency while using a mobile-based health application by Vagefi et al. [22]. In this study, 77.3% of initial registrants for One Health continued to enter PGHD and >96% of participants continued data entry after the first month. Clean, simple interphase and ease of navigation through the menus, for data entry in One Health, enhanced continued use. Bhuvan et al. identified health status tracking as a factor for continued use of the application in 47% [23]. One Health aimed at providing a platform for tracking health status by various inputs such as measurement of vital status, calorie intake, and physical activity.

In a 30-month study, Lee et al. reported the average duration of use of mobile-based health applications to be 25.62 weeks [24]. They also identified increased frequency of self-monitoring as a threat to continued use. In One Health, the frequency of PGHD entry by participants was monitored for only six months. The average number of monthly entries (for all variables combined) was 211. Motivation to continue data entry initially reduced but increased after four months. This pattern could be associated with the self-monitoring functionality of One Health. Most of the participants preferred entering health data twice a month.

Enhancement of self-care and status of control

Kao et al. demonstrated that individual self-care is the primary basis for designing telemedicine-based healthcare services [13]. In this study, the number of controlled hypertensives and diabetics, among One Health users was more compared to commencement levels. Mean values of SBP and HbA1c also decreased significantly. Wang et al. demonstrated the use of self-care behaviors in patients with chronic disease. Patients using mobile-based health applications increased the use of chronic disease management strategies (adjusted odds ratio (aOR) = 2.59) [25]. Wulfovich et al. reported that mobile health applications, enable better self-management and patient well-being [26]. One Health enhanced health promotion and promoted healthy behavior by prompting its users to enter PGHD.

Bengtsson et al. [27] demonstrated that an interactive mobile application was able to reduce values of SBP (mean change of 7 mmHg) and DBP (mean change of 4.9 mm Hg) significantly in an interventional study for eight weeks. In the current study reduction of DBP (0.60 (±2.31) mm Hg) alone was noted. The sample size was also different. There was no active intervention and observation was for six months. Zhai and Yu [17] demonstrated a statistically significant reduction over six months in HbA1c values when diabetes self-management mobile applications were used. In the current study, a significant mean reduction of 0.657 (±1.808) units of HbA1c was noted.

There are several challenges in delivering primary healthcare in India, from patients’ and providers’ sides alike [28,29]. Digital health interventions show potential for the management of primary healthcare, particularly in rural and health access-limited areas of LMICs, including India [30]. Digital health interventions in the form of text messages have been designed and delivered in rural India to reduce smoking [31]. One Health demonstrated the acceptability of mobile applications for health management among hypertensives and diabetics, One Health was also used for six months by self-motivated participants. Secondly, One Health also performed well in the collection of monthly follow-up data. Although the data were not according to pre-determined frequency, collection of this volume of PGHD demonstrated ease of use and data entry on One Health.

With the increasing use of DH in India and the provision of universal health coverage by digital technology-based programs, such as Rashtriya Swasthya Bima Yojana (RSBY) [32], it has become necessary to integrate mobile-based health applications into primary healthcare. One Health is an application developed for monitoring healthy behaviors and self-monitoring. One Health shows the potential of being integrated into primary healthcare for self-monitoring and enhancing self-motivation.

With regard to the strengths of the study, we used an integrated approach toward achieving glycemic and hypertensive control. Patients were monitored in multiple aspects such as blood glucose, blood pressure, diet, exercise, and medication adherence. As it was PGHD, patients were more involved in their self-care and were actively learning health care for the benefit of the patient, health care provider, and the health care system. The One Health app has been meticulously designed to enable patients to comprehensively monitor their health parameters while simultaneously receiving treatment from healthcare providers.

Limitations of the study

The study was conducted in central India and thus, cannot be generalized to other parts of India. India being a large country, has different patterns of utilization of telecommunication services. Hence, the authors express a need for careful consideration of the results for other geographical regions of India and elsewhere. Age- and sex-standardized rates for the usage of the application could have been added to understand the usage pattern of the application. Lack of identification of socio-economic variables for willingness and sustained use of the application. Follow-up of participants/patients beyond six months to identify drop-out and sustained use.

## Conclusions

The research herewith is the initial proof that mobile-based applications can be used for PGHD of NCD patients in central India and can be extended elsewhere. A mere provision of such applications increases the healthy behaviors in NCD patients. Such applications provide another horizon for healthcare interventions for patients. Inclusion of health prompts, physician feedback, and other interventions can be incorporated and evaluated for feasibility and effectiveness. Future research must explore and innovate the potential of digital healthcare in low-resource third-world countries.

## Appendices

Definition of the indicators used for the analysis of data

Completeness of Data Entry at Registration

Completeness of data upon registration indicates ease of usability of the application.

Socio-demographic details, blood pressure, and Random Blood Glucose levels were considered for completing the data entry at registration.

Completeness of Follow-Up

Completeness was assumed upon entering the minimum required number of all the identified variables. For blood pressure and blood glucose, the minimum requirement of entries was once a week. For HbA1c, the requirement for completed entries was once three months after the first entry during registration. For physical activity, the minimum requirement of consideration of completion was once a week. And, for diet and medications, daily entries were required to be considered complete. Several participants completing follow-up as per criterion are described in the results section.

Completeness of Data Entry at Each Follow-Up

Follow-up was considered monthly. Monthly entries were considered to be complete if, the number of minimum required entries for each of the identified variables were entered in the frequency mentioned previously.

Status of Control of Clinical Parameters at Baseline and End-Line

The status of control of blood pressure and blood glucose levels were considered as per the standard guidelines, referred to above. The number of patients with controlled blood sugar, HbA1c and blood pressure at baseline and end-line were compared.

Measurement of Various Indicators

For physical activity, the average number of calories burned by the total participants was monitored across the months.

For the diet data, the average total daily calorie intake was calculated and meals with the highest calorie intake during the day were identified. Both these variables were monitored across the months.

The frequency of data entry was measured for all the variables and monitored across the months. Number of entries was categorized across the number of days for which entries were made and reported.

One month was taken as the unit for follow-ups (monthly follow-up) for all indicators, but HbA1c. One month was defined as the 30 days after registration of the participant on the application. The mean frequency of data entry was calculated per month by averaging the total number of entries by the number of participants making entries in the past 30 days.
